# A Comparison of the Efficacy of Electroconvulsive Therapy and Transcranial Magnetic Stimulation in the Treatment of Depressive Disorder – Is One Better Than the Other?

**DOI:** 10.1192/bjo.2025.10140

**Published:** 2025-06-20

**Authors:** Zara Thurstan, Declan Hyland

**Affiliations:** ^1^University of Liverpool, Liverpool, United Kingdom; ^2^Mersey Care NHS Foundation Trust, Liverpool, United Kingdom

## Abstract

**Aims:** Major depressive disorder (MDD) and treatment-resistant depression (TRD) are significant public health challenges that not only severely affect quality of life but also place a substantial burden on healthcare systems. Individuals with TRD are at an increased risk of suicide, making early intervention essential.

Two prominent neuromodulation treatments, electroconvulsive therapy (ECT) and transcranial magnetic stimulation (TMS), are commonly used in clinical practice. Despite numerous studies comparing these interventions, definitive conclusions regarding their relative efficacy in treating depression are still lacking. This systematic review aims to compare the antidepressive effects of ECT and TMS, as measured by the Hamilton Depression Rating Scale (HAM-D), whilst also evaluating their clinical feasibility, tolerability, and patient acceptability to guide evidence-based treatment decisions.

**Methods:** A systematic literature review was conducted using the PubMed and Scopus databases, using keywords “TMS AND depression”, “ECT AND depression”, and “TMS OR ECT AND depression”. Out of 170 initially retrieved articles, six studies met the inclusion criteria of directly comparing the effects of ECT and TMS in patients diagnosed with MDD or TRD. Three other studies were also included in the analysis for their valuable insights on related outcomes.

**Results:** The findings suggest that ECT has a superior short-term antidepressive effect compared with TMS. Specifically, ECT achieved a response rate of 64.4% and remission rate of 53%, whereas high frequency rTMS (HFrTMS) yielded response and remission rates of 48.7% and 32.2%, respectively. Low frequency rTMS (LFrTMS) demonstrated a notably lower response rate of 20%. In cases of psychotic depression, ECT led to a 58.8% reduction in HAM-D scores, whilst TMS produced a 38% reduction. However, bilateral rTMS (BL-rTMS) emerged as a promising option, offering moderate efficacy alongside superior tolerability and lower dropout rates.

**Conclusion:** Whilst ECT remains the most effective short-term treatment for depression, its need for general anaesthesia and seizure induction may limit its acceptability. Despite being considered safe and effective by many psychiatrists, the stigma surrounding ECT creates a potential barrier to its widespread acceptance. TMS, particularly BL-rTMS, presents a less invasive alternative with better tolerability, making it a potentially reasonable option for patients and clinicians. These findings highlight the importance of personalised treatment plans that consider clinical severity, patient preference, and available resources. Future longitudinal studies are needed to assess long-term outcomes and adverse effects, such as the mild memory loss associated with ECT in the long term, to further refine the role of these neuromodulation therapies in managing MDD and TRD.

